# Risk factors of malnutrition in children with congenital heart disease: a meta-analysis

**DOI:** 10.3389/fped.2024.1258725

**Published:** 2024-07-29

**Authors:** Chen Zhang, Banghong Xu, Cuiying Zhu, Kai Pu, Lanzheng Bian

**Affiliations:** Department of Cardiothoracic Surgery, Children’s Hospital of Nanjing Medical University, Nanjing, China

**Keywords:** malnutrition, children, congenital heart disease, care, paediatric, management

## Abstract

**Background:**

The associated factors of malnutrition in children with congenital heart disease (CHD) must be evaluated to provide evidence for the treatment and care of such children.

**Methods:**

Two investigators searched the PubMed database until 25 June 2023 for literature about the associated factors of malnutrition in children with CHD. A meta-analysis of associated factors of malnutrition was performed by RevMan 5.3 software.

**Results:**

Thirteen studies involving 8,031 children with CHD were included. Pulmonary hypertension (OR = 3.81, 95% CI: 2.46–4.12), low birth weight (OR = 2.69, 95% CI: 1.25–5.77) and parents’ height (OR = 2.15, 95% CI: 1.89–2.92) were the associated factors of growth retardation (all *P* < 0.05). Pulmonary hypertension (OR = 3.77, 95% CI: 3.13–4.24), low birth weight (OR = 3.04, 95% CI: 2.61–4.18) and pneumonia (OR = 2.35, 95% CI: 2.08–2.83) were the associated factors of low body weight of children with CHD (all *P* < 0.05).

**Conclusions:**

Medical staff should fully understand the risk factors, strengthen nutritional support and enhance nursing care for children with CHD to reduce malnutrition.

## Background

Congenital heart disease (CHD) is the disorder or abnormal development of the heart and large blood vessels caused by various factors during embryonic development, and it leads to abnormal anatomical structure of the heart and/or large vessels (1). CHD occurs in about 0.8% of newborns (2). Most children with CHD need interventional or surgical treatment, and their nutritional status directly affects perioperative complications and postoperative recovery (3). Children with CHD are likely to be complicated with infection, heart failure and hypoxemia, which can lead to malnutrition and growth retardation. As a result of changes in systemic nutritional supply caused by abnormal haemodynamics, increased energy demand caused by surgical stress and the use of positive inotropic drugs and insufficient energy supply caused by fluid restriction, malnutrition is common in children with CHD (4, 5). Studies (6–8) have shown that the incidence of acute and chronic malnutrition in children with CHD can be as high as 50%, which is higher than the average level of hospitalised children in the same age. Therefore, the treatments and nursing care of malnutrition in children with CHD are critical.

The relevant guidelines (9, 10) define child malnutrition as an imbalance between nutritional needs and intake, resulting in insufficient accumulation of energy, protein or micronutrients, which has a negative impact on the growth and development of children. Malnutrition can increase the risk of postoperative infection in children with CHD, prolong mechanical ventilation and hospital stay, increase mortality and increase the economic burden of children's families (11–13). Therefore, the associated factors of malnutrition in children with CHD must be identified for targeted intervention to be carried out. At present, numerous studies have investigated the associated factors of malnutrition in children with CHD, but the sample size of most studies is small, and the factors included in these studies vary. Related meta-analyses to evaluate the associated factors of malnutrition in children with CHD are few. Therefore, this study analysed the related studies of malnutrition in children with CHD and systematically evaluated the associated factors of malnutrition in children with CHD to provide reference and evidence for the clinical treatment and nursing care of children with CHD.

## Methods

This meta-analysis was conducted and reported according to the Preferred Reporting Items for Systematic Reviews and Meta-analyses (PRISMA) statement (14).

### Bibliography retrieval

The two researchers searched the literature about the associated factors of malnutrition in children with CHD in PubMed, web of science, Embase, Cochrane Library, China National Knowledge Infrastructure (CNKI), Wanfang Database, Weipu and Chinese Biomedical Literature Database until 25 June 2023. The combination of subject words and free words was adopted. The search strategies were as follows: (“congenital heart disease” OR “congenital heart defects” OR “CHD”) AND (“malnutrition”) OR “nutrition” OR “weight” OR “retardation” OR “development”). We also reviewed and screened the references of included studies and related reviews to retrieve as many relevant studies as possible.

### Literature inclusion and exclusion criteria

The inclusion criteria of this study were as follows: the type of study was a case–control study, cohort study or cross-sectional study; the study population were children with CHD; the age was less than 18 years old; and the exposure factors were related to malnutrition in children with CHD. The outcome index was malnutrition, the definition of malnutrition was based on the growth standard Z score established by World Health Organization (WHO) (15) and Z score < −2 was defined as malnutrition. The exclusion criteria of this study were as follows: literature with low quality or high bias risk (quality score < 4); literature that could not obtain full text or incomplete data; and repeatedly published studies.

### Quality assessment

The two researchers independently assessed the quality of the included studies, cross-checked after the evaluation and negotiated with a third researcher to resolve differences. The case–control study and cohort study were evaluated with the Newcastle–Ottawa scale (16), which consists of eight items. The literature was evaluated by the semi-quantitative principle of star system, with a total score of 9 points and a high-quality study ≥6. The cross-sectional study was evaluated using the bias risk assessment criteria recommended by the American Institute for Health Care quality and Research (17). A total of 11 items were answered with “yes”, “no” and “unclear”. The total score was 11; ≤3 indicates low quality, 4–7 indicates medium quality and ≥8 indicates high quality.

### Statistical method

In this study, RevMan 5.3 software was used for statistical analysis of the data. The effect was evaluated by odds ratio (OR) and its 95% confidence interval (CI). The included studies were analysed for clinical heterogeneity. Chi-square test combined with I^2^ was used to quantitatively assess the heterogeneity. If *P* ≥ 0.1, I^2^ < 50%, then there was no heterogeneity among studies, and the fixed-effect model was used. If *P* < 0.1, I^2^ ≥ 50%, then there was heterogeneity among studies, and the random-effect model was used. If clinical heterogeneity was too large, descriptive analysis was used. Sensitivity analysis was performed to test the stability of the results. In this study, the difference was statistically significant when *P* < 0.05.

## Results

**Figure 1:**
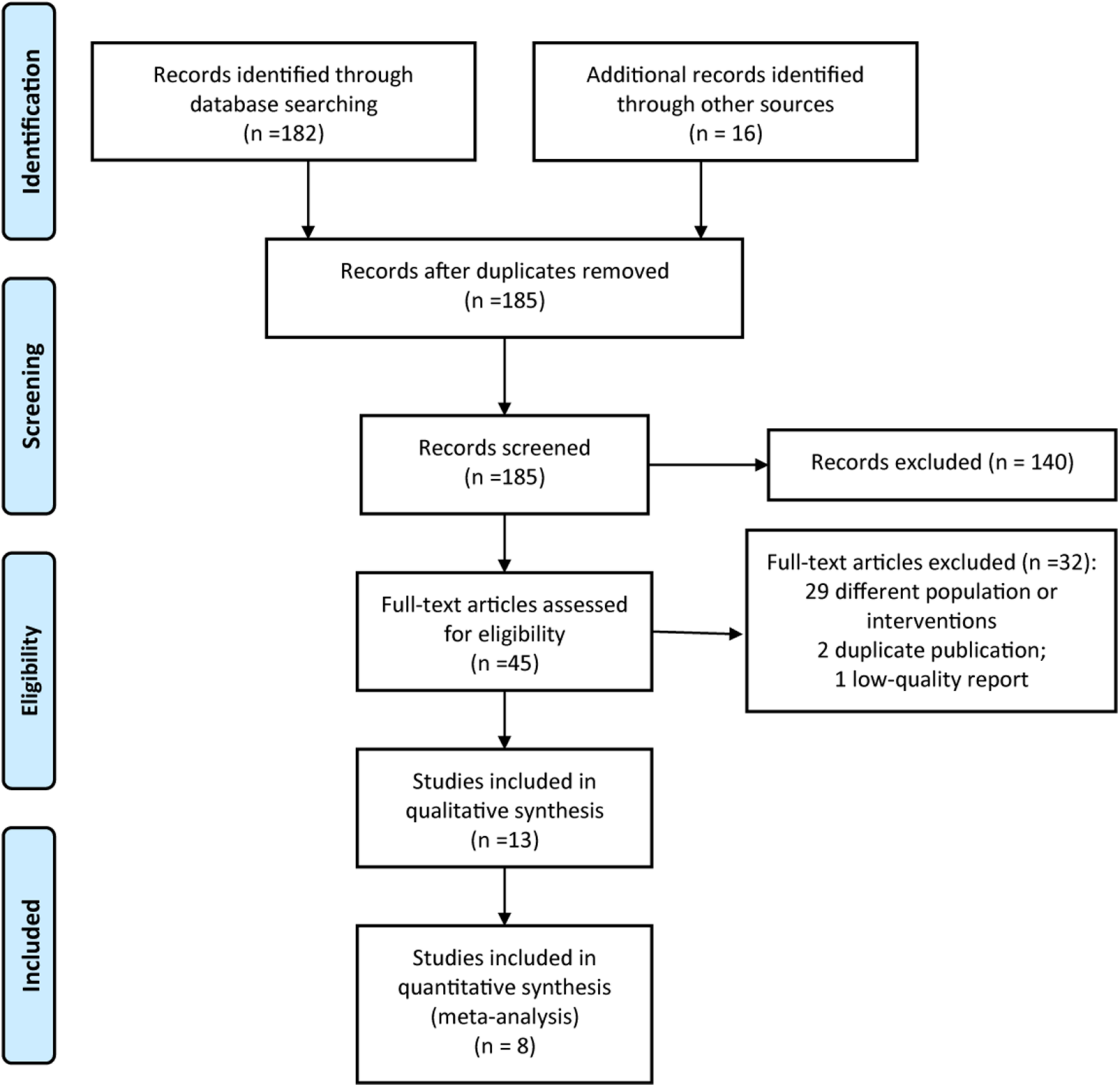
PRISMA flow diagram of study selection.

We initially identified 198 related reports, and we screened the remaining 185 articles after duplicate removal. After reading the titles and abstracts and removing the articles that obviously did not meet the inclusion criteria, we then read the full text and finally included 13 reports (18–30). The 13 studies (18–30) involved a total of 8,031 children with CHD. The literature screening process is shown in Figure 1, and the basic characteristics of the literature are shown in Table 1.

**Table 1:** Characteristics and quality of included studies.

Study	Country	Design	Sample size	Age	Influencing factors	Bias risk assessment score
Hu et al. 2021 (19)	China	Cohort study	312	2–12 years	Age, family income, types of congenital heart disease, anxiety level,	8
Huang et al. 2020 (20)	China	Cross-sectional study	334	4.3 ± 1.9 years	Pulmonary hypertension, disease, birth weight, pneumonia, heart failure	5
Jiao 2012 (21)	China	Cross-sectional study	143	11.4 ± 6.6 months	Pulmonary hypertension, birth weight, age, number of other malformations, feeding difficulties, intake, maternal access to nutritional knowledge, maternal feeding behaviour	9
Li et al. 2020 (22)	China	Cross-sectional study	880	0–24 months	Gender, age, types of congenital heart disease	6
Monteiro et al. 2012 (23)	Brazil	Cross-sectional study	132	9.43 ± 6.08 months	Age, gender, height, Apgar score, thickness of subscapular skinfold	7
Okoromah et al. 2011 (24)	Nigeria	Case–control study	149	3–192 months	Birth weight, congestive heart failure, modified Ross score, age, sex, type of congenital heart disease, arterial oxygen saturation, haemoglobin, intake, birth order, age of milk weaning, social status	6
Qin 2017 (18)	China	Case–control study	1,384	2–4 years	Mother's anxiety, mother's depression, mother's perception of disease, mother's disease knowledge, mother's education level.	6
Vaidyanathan et al. 2009 (25)	India	Cohort study	476	15.2 ± 16.2 months	Birth weight, age of operation, height of parents, marriage of next of kin.	6
Wang 2012 (26)	China	Cross-sectional study	354	6.53 ± 3.22 months	Pulmonary hypertension, birth weight, pneumonia	7
Xie et al. 2020 (27)	China	Cross-sectional study	112	2.82 ± 0.81	Birth weight, maternal anxiety, maternal depression, maternal perception of disease	6
Xu 2019 (28)	China	Cross-sectional study	160	3.72 ± 1.42	Mother's disease cognition and anxiety	6
Zhang et al. 2020 (30)	China	Cohort study	3,165	1–3,671 days	Pulmonary hypertension, cyanotic congenital heart disease, age, mechanical ventilation, RACHS-1, parental height, single ventricle, residual shunt, long-term use of diuretics, Ross score	7
Zhang et al. 2022 (29)	China	Cross-sectional study	430	0–5 years	Heart malformation type, mechanical ventilation time, intensive care time, hospital stay	7

RACHS-1, risk adjustment for congenital heart surgery 1.

Eight cross-sectional studies were included in this study. The score of bias risk assessment was 510, of which five studies carried out quality control on the measurement of outcome indicators, and four studies explained the reasons for excluding analysis to be included in the study. Only two studies summarised the response rate of patients and the integrity of data collection. Three cohort studies were included, and the score of bias risk assessment was 7–8. Three case–control studies were included, in which one study did not explain the method of determining the case group and whether the response rate of the case group and the control group was the same, and the two other studies did not explain whether the response rate of the case group and the control group was the same.

As shown in Table 2, several studies reported the associated factors of growth retardation of children with CHD. This meta-analysis indicated that pulmonary hypertension (OR = 3.81, 95% CI: 2.46–4.12), low birth weight (OR = 2.69, 95% CI: 1.25–5.77) and parents' height (OR = 2.15, 95% CI: 1.89–2.92) were the associated factors of growth retardation of children with CHD (all *P* < 0.05).

**Table 2:** Meta-analysis on the associated factors of growth retardation of children with congenital heart disease.

Factors	Number of included studies	Heterogeneity	Model for meta-analysis	OR	95% CI	*P*
Pulmonary hypertension	3	45%	Fixed	3.81	2.46–4.12	0.002
Low birth weight	2	60%	Random	2.69	1.25–5.77	0.014
Parents’ height	3	41%	Fixed	2.15	1.89–2.92	0.033

As shown in Table 3, several studies reported the associated factors of low body weight of children with CHD. This meta-analysis indicated that pulmonary hypertension (OR = 3.77, 95% CI: 3.13–4.24), low birth weight (OR = 3.04, 95% CI: 2.61–4.18) and pneumonia (OR = 2.35, 95% CI: 2.08–2.83) were the associated factors of low body weight of children with CHD (all *P* < 0.05).

**Table 3:** Meta-analysis on the associated factors of low body weight of children with congenital heart disease.

Factors	Number of included studies	Heterogeneity	Model for meta-analysis	OR	95% CI	*P*
Pulmonary hypertension	4	36%	Fixed	3.77	3.13–4.24	0.017
Low birth weight	4	11%	Fixed	3.04	2.61–4.18	0.009
Pneumonia	3	65%	Random	2.35	2.08–2.83	0.042

Some studies did not explain the type of malnutrition involved, so we could not combine those data for analysis. Among them, the higher the Ross score of heart failure in children with CHD, the higher the risk of malnutrition. Some studies also reported that heart failure is the associated factor of malnutrition in children with CHD. Besides, age, gender, feeding difficulties, social status, postoperative residual shunt and long-term oral diuretics all affect the nutritional status of children with CHD.

We excluded each of the results of the meta-analysis one by one, and the results of the meta-analysis did not change significantly. The results of sensitivity analysis suggested that the results of the meta-analysis had good stability. Limited by the number of included studies, we could not perform the funnel plots. Regression analyses on those synthesised outcomes showed no publication biases (all *P* > 0.05).

## Discussion

Children with CHD are prone to malnutrition because of increased energy consumption and energy utilisation disorders, and postoperative complications and mortality in children with malnutrition are higher than those in children with normal nutritional status (31). Among 89 hospitalised children with CHD aged 12–45 months, 65.2% children have different degrees of malnutrition, and poor cardiopulmonary function is a common associated factor of malnutrition in children with CHD (32). Cardiac dysfunction and pneumonia lead to increased catecholamine secretion, increased work done by respiratory muscles, increased resting energy consumption and increased demand for nutrition (33). On the one hand, if complicated with pulmonary hypertension or heart failure, CHD will cause venous blood stasis and gastrointestinal dysfunction, affecting digestion and absorption; on the other hand, CHD will cause insufficient tissue perfusion, tissue ischemia and hypoxia and limited nutrition utilisation (34). The liquid intake of children is usually limited to reduce the cardiac volume load, further hindering the intake of nutrition (35). The interaction of the above factors leads to malnutrition in children with CHD. Therefore, timely treatment of the primary disease and active prevention of complications are the basis for improving malnutrition in children with CHD. This study analysed the associated factors of malnutrition in children with CHD. Clinical medical workers should adopt early warning and nursing measures for children with these risk factors to reduce the incidence of malnutrition.

The possible mechanisms of CHD affecting growth, development and nutritional status should be considered. Intestinal dysfunction due to venous congestion caused by heart failure leads to digestive and absorption disorders; at the same time, cardiac output decreases during heart failure, resulting in insufficient blood supply to the systemic circulation, hypoxia and acidosis in the surrounding tissues and nutritional utilisation disorders (36). Children with CHD are often complicated with purple, pulmonary hypertension and congestive heart failure. The more serious the pulmonary hypertension is, the longer the course of disease is, the greater the effect on growth and development will be (33, 37). Pulmonary hypertension can cause increased right ventricular afterload and pulmonary vasoconstriction, resulting in hypoxia and right heart failure, insufficient tissue oxygen supply, disturbance of nutritional utilisation and intestinal venous congestion (38, 39). These factors may increase the risk of poor growth and malnutrition in children. The effects of these factors on growth and development must be evaluated, because improving the state of malnutrition is beneficial to postoperative rehabilitation and reducing the risk of surgical complications and perioperative mortality.

Different from disease factors, congenital or genetic factors will affect the nutritional status of children with CHD for a long time, and preoperative malnutrition has a greater impact on weight than on height. Through surgical treatment, the abnormal anatomical structure of the heart can be corrected, and the timely supply of adequate nutrition after operation can help the children improve malnutrition (40). Studies (41, 42) have shown that low birth weight newborns often require 1 to 2 years to “catch up” to their peers. With the progress of medical technology, the age of children with CHD receiving surgical treatment is becoming increasingly younger. If children are born with a low weight, they may not be able to catch up with their growth in a short time, so they are characterised by growth retardation. In addition, height is a polygenic trait, and a child's height is affected by genetic factors. For tall parents with offspring who may also be taller than their peers, coupled with the causes of disease, their children are highly likely to develop growth retardation (43). Children with CHD with low birth weight and low parental height have low growth potential and are more likely to develop stunting in the long term than their counterparts. In clinical practice, attention should be paid to the identification of such children, and guidance and intervention to their families should be given if necessary.

Family factors are the associated factors of malnutrition in children with CHD. Given the particularity of children's age, parents play an important role in children's disease management. Many studies have shown that family economic status, parents' education level and parents' feeding behaviour are the associated factors of poor nutrition in children with CHD (44). Parents’ correct perception of disease is the protective factor of malnutrition in children with CHD, whereas parents' anxiety and depression will increase the risk of malnutrition (45). The caregivers' incorrect perception of the disease will lead to negative mood, incorrect diet structure and forced eating behaviour (46). Whilst paying attention to the children, the clinical medical staff should also pay close attention to the caregivers and guide the caregivers to adopt scientific and reasonable feeding behaviour.

## Conclusions

In conclusion, many factors are associated with malnutrition in children with CHD, amongst which pulmonary hypertension and low birth weight are the most common factors. In addition, the cardiopulmonary function of the children and the disease cognition and emotion of the caregivers are common associated factors. The limitations of this meta-analysis were the wide age range of the studied population, different types of CHDs and no heterogenicity between the studied groups. Clinical health care providers should prioritise the prevention and active intervention of children with CHD, such as provide timely treatment of primary diseases, improve caregivers' awareness of the disease, alleviate their negative emotions and promote them to adopt correct feeding behaviour to reduce the risk of malnutrition in children with CHD.

## Data Availability

The original contributions presented in the study are included in the article/Supplementary Material, further inquiries can be directed to the corresponding authors.
